# Routine daily physical activity and glucose variations are strongly coupled in adults with T1DM

**DOI:** 10.14814/phy2.12644

**Published:** 2015-12-10

**Authors:** Sarah S. Farabi, David W. Carley, Ali Cinar, Lauretta Quinn

**Affiliations:** ^1^ Center for NarcolepsySleep and Health ResearchUniversity of Illinois at ChicagoChicagoIllinois; ^2^ Department of Biobehavioral Health ScienceUniversity of Illinois at ChicagoChicagoIllinois; ^3^ Department of MedicineUniversity of Illinois at ChicagoChicagoIllinois; ^4^ Department of BioengineeringUniversity of Illinois at ChicagoChicagoIllinois; ^5^ Ilinois Institute of TechnologyChicagoIllinois

**Keywords:** Continuous glucose monitoring, daily activity, glucose variations, type 1 diabetes

## Abstract

Type 1 Diabetes (T1DM) is characterized by altered glucose homeostasis resulting in wide glucose variations throughout a 24‐h period. The relationship between routine daily physical activity and glucose variations has not been systematically investigated in adults with T1DM. The objectives of this study were to characterize and quantify the relationship between routine daily activity and glucose variations in a small group of adults with T1DM. Adults with T1DM treated with an insulin pump were recruited for the study. Over a 3‐day period, glucose variations were monitored with a continuous glucose monitoring system (CGMS) and routine daily physical activity was assessed using an accelerometer‐based physical activity‐monitoring band. Simultaneous glucose and physical activity data for one 24‐h period were used for analysis. Cross‐correlation function and wavelet coherence analyses were employed to quantify the coupling between physical activity and glucose. Twelve subjects were included in the analysis. Cross‐correlation function analysis revealed strong coupling between activity and glucose. Wavelet Coherence demonstrated that slower oscillations (120–340 min) of glucose and physical activity exhibited significantly greater coherence (*F* = 12.6, *P* < 0.0001) than faster oscillations (10 and 120 min). Physical activity and glucose demonstrate strong time and frequency‐dependent coupling throughout a 24‐h time period in adults with T1DM.

## Introduction

Type 1 Diabetes (T1DM) is characterized by altered glucose homeostasis resulting in wide glucose variations throughout a 24‐h period. Achievement of adequate glycemic control in diabetes has been shown to play a critical role in delaying or preventing development of vascular complications associated with diabetes (Skyler 2009). The effects of structured physical activity (exercise) on glucose control in T1DM have been investigated with varying results (Yardley et al. 2014). Most studies of this interaction have been conducted under controlled laboratory settings, which can be much different than real‐life (Zecchin et al. 2013). There has been much less investigation of the relationship between routine daily physical activity (as opposed to structured exercise regimens) and real‐time glucose variations in real‐life settings. Actigraphy monitors make it possible to measure physical activity and continuous glucose monitoring systems (CGMS) can provide increasingly accurate glucose levels in real‐world settings. Furthermore, the nature of the relationship between routine daily physical activity and glucose variations may vary throughout the 24‐h period, and these systems can record over periods of several days. Defining temporal variations in coupling between glucose and routine daily physical activity may be important in improving glucose control in individuals with T1DM. We hypothesized that the coupling between glucose and activity in a 24 h period would exhibit different characteristics based on time and fluctuation frequency. The objectives of this study were to determine the relationship between routine daily physical activity and glucose variations in adults with T1DM.

## Methods

### Subjects and protocol

Adults with T1DM treated by continuous subcutaneous insulin infusion were included in this analysis, which is part of a larger study for which data were collected from 2010 to 2011. After obtaining initial informed consent, hemoglobin A1c (HbA1c) was measured and a brief physical examination was carried out. A CGMS sensor was inserted into the subcutaneous tissue in the abdominal area and connected to the blinded recorder (iPro2, Medtronics, Northridge, CA). This unit provided a time‐stamped average interstitial glucose value every 5 min. A physical activity‐monitoring band (SenseWear Pro3^™^ Armband; BodyMedia, Inc. Pittsburgh, PA) was placed on the upper aspect of the nondominant arm. General level of physical activity was assessed by self‐report with two questions: “How would you describe how physically active you are: vigorous, moderate or sedentary?” and “How many days per week do you engage in physical exercise for at least 60 min?” Subjects then left the laboratory and were free to go about usual life routines for 3 days. For purposes of the analyses reported here, glucose and physical activity data for a 24‐h period, beginning at 1900 and ending at 1900 the following day, were utilized. For each subject, the 24‐h period with the fewest missing data were used for analysis. For missing data, the value at the last point of nonmissing data were carried forward until the next nonmissing point. One (of the 12) subjects had 35 min (7 points) of missing glucose data at last 35 min of the recording period. For activity, five subjects had missing data which ranged from 1 min to 21 min in length (mean 7.8 min). Physical activity counts (peaks) were recorded every minute and the moving average (5‐min window) of physical activity counts was resampled every 5 min over the 24‐h period to allow alignment with glucose values. All study procedures were approved by the Institutional Review Boards of the University of Illinois at Chicago and Illinois Institute of Technology.

### Statistical measures

#### Cross‐correlation function

Cross‐correlation analysis determines the relationship between two time‐series waveforms by applying a lag to one of the waveforms. The cross‐correlation function extends simple correlation computation by identifying the best‐fit lag. Identification of a consistent delay between two waveforms can support causal inferences as a physical effect cannot precede it's cause. We calculated the cross‐correlation function between glucose and physical activity for each 24‐h recording applying lags of −500 min to 500 min to glucose values. Eleven of the 12 subjects exhibited a dominant peak correlation at a lag between −100 min and 100 min. Therefore, we identified the first peak in the correlation (i.e. with the lowest lead or lag value) between the two signals for each subject.

#### Wavelet coherence

Cross‐correlation analysis, while useful for identifying a possible fixed delay between two coupled processes, is insensitive to possible differences in coupling for fluctuations of differing frequencies or to variations in coupling over time. As described by Grinsted et al. (2004), wavelet coherence analysis complements correlation analysis: it identifies time varying and frequency specific coupling, but is not as useful for identifying a fixed delay between two systems or signals (Grinsted et al. 2004). The theory underlying wavelets has been reviewed elsewhere (Islam 2011) and is beyond the scope of this paper. Briefly, wavelets are mathematical functions that can be used to decompose the original physical activity and glucose waveforms into different frequency components and then compute the time varying coherence between them at each underlying frequency (Grinsted et al. 2004). As recommended by Grinsted et al. (2004), we utilized the Morlet wavelet function for the present analysis (Grinsted et al. 2004). This yielded updated coherence values every 5 min for each of 68 underlying oscillations with periods ranging from 10 min to 340 min. To facilitate analysis, we computed the average coherence within each recording for each of five Bands with differing period ranges (Band 1: 10–30 min; Band 2: 30–60 min; Band 3: 60–120 min; Band 4: 120–240 min; and Band 5: 240–340 min). For each time point in each Band, the computed coherence value was analogous to the squared correlation coefficient (*r* ^2^) for that time and Band. Thus, the analysis computed the time‐varying and frequency specific coupling between daily physical activity and glucose variations. In order to identify which computed coherence values were statistically significant (*P* < 0.05), we utilized Monte Carlo simulations (*N* = 500) as described by Grinsted and colleagues (Grinsted et al. 2004). The mean coherence, the number of intervals of significant coherence and the total duration of significant coherence were tabulated for each Band of each recording. Coherence analysis also reveals the phase relationship between the two processes at each time point in each Band. To be able to more logically interpret this information, the phase was converted to an equivalent delay for that time and Band. ANOVA was used to identify differences in coherence parameters among the Bands. Pairwise differences between Bands were determined by post‐hoc tests using Scheffe's test.

#### Bivariate correlation

The Pearson product‐moment correlation coefficient was determined for the mean coherence values in each Band in relation to HbA1c as a measure of average glycemic control.

## Results

### Demographics

Eighteen people completed the protocol; data from 12 subjects (5 male, 7 female) who had minimal missing data for glucose and activity for a 24 h period are included in this analysis. Data from six of the subjects started on day 1 of their recordings and data from six of the subjects started on day 2 of recording. As summarized in Table 1, the study group comprised young to middle aged nonobese adults with typical glucose control for individuals with T1DM. Subjects were quite active, reporting 4.7 ± 1.42 days of exercise per week, with a range of 3–7 days (Table 1). None of the subjects reported use of a personal CGMS.

**Table 1:** Descriptive statistics: data are reported as mean ± SD or total

Descriptive statistics (*n* = 12)	
Male	5
Age (years)	30.1 ± 13.7
A1c (%)	7.4 ± 0.7
Duration (years)	20.1 ± 14.0
BMI (kg/m^2^)	24.3 ± 5.3
Physical activity level (*n* = 10)	
Sedentary	0
Moderate	8
Vigorous	2
Frequency of weekly physical exercise (days)	4.7 ± 1.42

### Cross‐correlation

**Figure 1:**
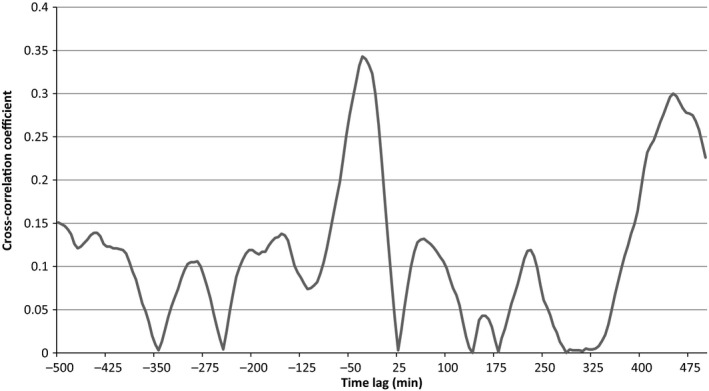
Example cross‐correlation function between physical activity and glucose. Demonstrates the cross‐correlation function for lags between −500 and 500 for one subject. The first peak is the highest correlation with several other peaks at varying lags.

Figure 1 demonstrates the cross‐correlation function between glucose and physical activity for one subject over lags from −500 to +500 min. As can be seen in Figure 1, the cross‐correlation showed periodic fluctuations and multiple peaks with the first peak being the highest and closest to zero. The average peak correlation for all subjects was 0.35 ± 0.14 at a mean lag of 13.4 ± 82.8 min, which was not significantly different from zero (*P* = 0.59).

### Coherence

**Figure 2:**
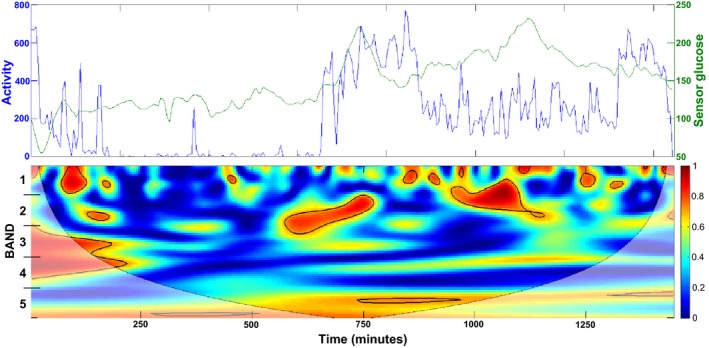
Coherence between routine daily physical activity and glucose variations. Raw physical activity and glucose sampled every 5 min for a 24‐h period (top panel). Heat map depicting frequency specific and time varying coherence between physical activity and glucose over 24 h. Red areas bounded by a black line indicate statistically significant coherence (*P* < 0.05), based on Monte Carlo simulation. Bands on the y axis indicate the cut‐points for the five frequency Bands with differing period ranges used in the analysis (Band 1: 10–30 min; Band 2: 30–60 min; Band 3: 60–120 min; Band 4: 120–240 min; and Band 5: 240–340 min).The lighter shaded area indicates the cone of influence (COI) or areas where the results of coherence cannot be trusted, only the values outside the COI (not shaded) were included in the analysis.

**Figure 3:**
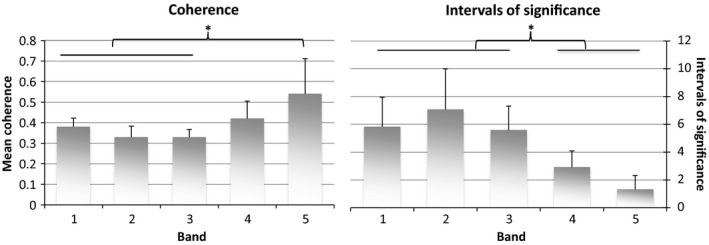
Mean coherence and intervals of significance by band. Individual bars represent mean ± SE. (*) signifies *P* < 0.05. Oneway ANOVA with Scheffe's test for multiple comparisons were used to determine differences between bands.

Figure 2 provides the raw activity and glucose data as well as the coherence between these two processes for one subject. This figure illustrates that coherence between routine daily physical activity and glucose variations varied according to both time and fluctuation frequency (Band). Bounded areas in red signify time and frequency specific intervals of statistically significant coherence between routine daily physical activity and glucose variations. One‐way ANOVA revealed that mean coherence was not equivalent across all Bands (Fig. 3), and post‐hoc contrasts demonstrated that slower oscillations (Bands 4 & 5) exhibited significantly greater coherence (*F* = 12.6, *P* < 0.0001) than the faster oscillations (Bands 1–3). In contrast, the number of intervals of significant coherence demonstrated the converse pattern (*F* = 18.17, *P* < 0.0001 by ANOVA); with more rapid fluctuations in Bands 1–3 exhibiting more intervals than slower fluctuations in Bands 4 and 5 (Fig. 3). The average percent of time spent in significant coherence was high among all bands (54.5 ± 3.5%, *P* < 0.0001 vs. zero); and did not differ between bands. The mean phase delay for all bands was 2.5 ± 3.5 min, which was not different from zero.

Bivariate Correlation demonstrated a significant negative association between mean coherence in Bands 1–3 and HbA1c (Band 1: *r* = −0.64, *P* = 0.03; Band 2: *r* = −0.76, *P* = 0.004; Band 3 = −0.64, *P* = 0.03). There was no association of HbA1c and mean coherence in Bands 4 & 5.

## Discussion

Our study represents a systematic evaluation of associations between routine daily physical activity and glucose variations in a small group of adults with T1DM in a free‐living, uncontrolled setting. Cross correlation analysis demonstrated that the coupling between glucose variations and routine daily physical activity was variable, with multiple peaks at multiple lags and coherence analysis showed that this coupling was both time and frequency dependent. The highest coherence was observed between slow fluctuations in glucose and physical activity, however, there were more intervals of statistically significant coherence between more rapid fluctuations. Subjects with higher coherence between rapid fluctuations also demonstrated significantly lower HbA1c values, suggesting potential clinical relevance for the coupling between daily physical activity and glucose. Understanding factors that influence glucose homeostasis is crucial to inform clinical management strategies for individuals with T1DM.

Structured physical exercise is well‐known to have positive effects on insulin sensitivity and glucose control in healthy adults as well as those with type 2 diabetes (Hawley and Lessard 2008). Although results of studies have been mixed and the approach has not been optimized, it does appear that exercise can impact glucose control in a clinically meaningful fashion in these individuals (Yardley et al. 2014). Much less is known about the continuous relationship between routine daily physical activity and glucose variations. Such knowledge may be important not only to inform routine clinical management of T1DM, but also to facilitate the evolution of closed loop systems for insulin administration. Zecchin et al. (2013) reported findings similar to ours; that physical activity measured by a physical activity monitoring system was correlated with glucose variations in a group of subjects with T1DM (Zecchin et al. 2013). Zecchin et al. utilized a laboratory setting, allowing for standardization of multiple factors potentially impacting the relationship between glucose variation and physical activity; such as insulin therapy, meals and intensity of physical activity. Our data were obtained in a real‐life setting, which is much more variable than an in‐laboratory controlled setting. In the present study, it is interesting to note that a significant coupling between physical and activity was observed despite the lack of control of these variables.

We demonstrate, in the present study, a strong coupling between minute‐to‐minute changes in physical activity and those in glucose level. Correlation analysis revealed significant coupling between glucose and daily physical activity but failed to identify a consistent global lead or lag between the two processes. It makes sense that, throughout a 24‐h period, the strength and even the nature of the relationship between activity and glucose is variable; with each process potentially leading or lagging the other at different times during the day. At some points, changes in glucose may elicit changes in physical activity. For example, an individual who is planning an exercise session may eat sugar to ensure that their blood sugar does not fall during or after exercise (Vanelli et al. 2006; Guelfi et al. 2007). In this instance, we would expect to see a rise in glucose prior to a rise in the activity level. Conversely, changes in activity also can precipitate changes in glucose; high levels of activity may elicit an increase in insulin sensitivity driving glucose levels lower (Yardley et al. 2013; Silveira et al. 2014). Furthermore, the relationship between glucose and activity likely differs between sleep and wake. Future studies should employ both food and sleep diaries to examine the impact of these factors. Due to this range of potential interactions between glucose and activity, we examined both positive and negative lags using cross correlation analysis. The fact that the average lag between glucose and activity was close to zero with a high standard deviation suggests that the interaction between glucose variations and physical activity is indeed bidirectional and that over a 24 h period each processes leads the other during some periods of the day.

Coherence analysis also revealed strong coupling between routine daily physical activity and glucose variations (mean coherence values ranged between 0.35 and 0.60). Wavelet coherence can appropriately be viewed to provide an experimental estimate of the frequency specific and time varying coupling between two systems or processes, with the coherence value being analogous to the squared correlation, *r* ^2^ (Grinsted et al. 2004). It makes physiologic sense that physical activity would impact glucose levels in individuals with T1DM. Indeed, structured physical activity has been shown to lead to both hypo‐ and hyperglycemia in people with T1DM depending on both the type and duration of the physical activity (Marliss and Vranic 2002; Camacho et al. 2005). Our findings are consistent with these observations; with slow fluctuations in routine daily physical activity and glucose exhibiting higher mean coherence than more rapid fluctuations: 0.41–0.53 versus 0.32–0.38, respectively (*P* < 0.05; Fig. 3). This suggests that slow or sustained changes in physical activity, lasting at least one hour, are more consistently accompanied by parallel changes in glucose than are rapid or transient changes in physical activity.

The results of our current study do not permit us to definitely determine causality. Despite the experimental evidence to support that physical activity influences glucose levels, it is also likely that glucose levels can influence physical activity. This is especially plausible during the sleep period. Pillar et al. (2003) showed that rapid changes in glucose were associated with increased awakenings from sleep in a group of children with T1DM (Pillar et al. 2003). Our findings are also consistent with this finding. As illustrated in Figure 2, nocturnal bouts of physical activity, most probably associated with arousal or awakening, tended to be of rapid onset and transient; on the order of seconds to minutes. In association with such events, we often observed brief intervals of significant coherence between physical activity and glucose level in Bands 1 and 2 (periods of 10–60 min). Although there is a sleep detection component to the armband, a recent study showed that different ambient temperatures can affect the reliability of measurement of sleep parameters, especially sleep onset latency (Shin et al. 2015). The timing of sleep in our study may be biased as we do not have sleep diaries from subjects recording the timing of sleep. Thus, we are limited in assessment to nocturnal activity (in which we expect that the subjects were sleeping).

The clinical and biological implications of the observed time and frequency‐specific coupling between daily physical activity and glucose also cannot be determined from this present study. The potential relevance, however, is suggested by our observation that mean coherence between rapid fluctuations in routine daily physical activity and glucose variations (Bands 1 and 2) were significantly associated with HbA1c levels in this small group of individuals with T1DM. Individuals who had good glucose control (lower HbA1c) demonstrated higher mean coherence than those with poor glucose control. To the extent that the coherence levels observed here during a single 24‐h period for each subject were representative of average long‐term coherence between glucose and physical activity for that subject, understanding the basis of the observed correlation with HbA1c may have important clinical implications.

Defining the relationship between glucose and physical activity in people with T1DM is of great importance. In the present study, we highlight the strengths of utilizing coherence analysis in defining the time and frequency dependent nature of this relationship. However, the findings of the current study are limited by a single day of recording with a small number of subjects. Future studies with more subjects, longer recording times and with control of variables such as food and insulin timing in real life settings, and using wavelet coherence for analysis to quantify glucose/activity coupling, are needed to confirm and expand the present findings.

In summary, this study presents a systematic evaluation of associations between routine daily physical activity and glucose variations in adults with T1DM. Strong time and frequency‐dependent coupling between these two processes was demonstrated using both cross‐correlation function and wavelet coherence analyses. The association between coherence and HbA1c suggests a direct clinical relevance of coupling between daily routine physical activity and glucose variations, but determining the true implications and mechanisms underlying these associations need to be investigated in future research.

## Conflict of Interest

None declared.
